# Rapid Identification and Multiple Susceptibility Testing of Pathogens from Positive-Culture Sterile Body Fluids by a Combined MALDI-TOF Mass Spectrometry and Vitek Susceptibility System

**DOI:** 10.3389/fmicb.2016.00523

**Published:** 2016-04-20

**Authors:** Yueru Tian, Bing Zheng, Bei Wang, Yong Lin, Min Li

**Affiliations:** ^1^Department of Laboratory Medicine, Shanghai Medical College, Huashan Hospital, Fudan UniversityShanghai, China; ^2^Department of Laboratory Medicine, Renji Hospital, Shanghai Jiaotong University School of MedicineShanghai, China

**Keywords:** MALDI-TOF mass spectrometry, Vitek AST system, sterile body fluids positive culture, rapid diagnosis, clinical impact

## Abstract

Infections of the bloodstream, central nervous system, peritoneum, joints, and other sterile areas are associated with high morbidity and sequelae risk. Timely initiation of effective antimicrobial therapy is crucial to improving patient prognosis. However, standard final identification and antimicrobial susceptibility tests (ASTs) are reported 16–48 h after a positive alert. For a rapid, effective and low-cost diagnosis, we combined matrix-assisted laser desorption/ionization time of flight mass spectrometry with a Vitek AST system, and performed rapid microbial identification (RMI) and rapid multiple AST (RMAST) on non-duplicated positive body fluid cultures collected from a hospital in Shanghai, China. Sterile body fluid positive culture and blood positive culture caused by Gram negative (GN) or polymicrobial were applied to the MALDI–TOF measurement directly. When positive blood culture caused by Gram positive (GP) bacteria or yeasts, they were resuspended in 1 ml brain heart infusion for 2 or 4 h enrichment, respectively. Regardless of enrichment, the RMI (completed in 40 min per sample) accurately identified GN and GP bacteria (98.9 and 87.2%, respectively), fungi (75.7%), and anaerobes (94.7%). Dominant species in multiple cultures and bacteria that failed to grow on the routing plates were correctly identified in 81.2 and 100% of cases, respectively. The category agreements of RMAST results, determined in the presence of various antibiotics, were similarly to previous studies. The RMI and RMAST results not only reduce the turnaround time of the patient report by 18–36 h, but also indicate whether a patient's antibiotic treatment should be accelerated, ceased or de-escalated, and adjusted the essential drugs modification for an optimized therapy.

## Introduction

Infections of the bloodstream, central nervous system, peritoneum, joints, and other sterile areas are associated with high morbidity and risk of sequelae (Thigpen et al., 2011; Goto and Al-Hasan, 2013; Chon et al., 2014; Ascione et al., 2015; Bagheri-Nesami et al., 2015). Among these, bloodstream infection (BSI) is most serious, because it can rapidly deteriorate into sepsis, severe sepsis, or septic shock. BSIs have become a major cause of death in European intensive care units, incurring a mortality rate of 30–50% (Vincent et al., 2006). To improve this prognosis, timely initiation of effective antimicrobial therapy is essential (Kumar et al., 2006; Vincent et al., 2006; Dellinger et al., 2013; Chon et al., 2014; Ascione et al., 2015; Bagheri-Nesami et al., 2015). Currently, culture remains the gold standard of infection diagnosis. However, standard final identification (ID) and ASTs are reported 16–48 h after a positive alert. During this delay, the clinician must administer an empirical antimicrobial therapy, typically a broad-spectrum antibiotic or an antibiotic cocktail to cover all likely pathogens. However, inappropriate antimicrobial therapy will worsen the outcome (Ibrahim et al., 2000; Tumbarello et al., 2010; Cain et al., 2015). Moreover, the long-term use of dispensable broad-spectrum antibiotics promotes antibiotic resistance and spread, increases cost and lengthens hospital stays (Blot et al., 2002; Tumbarello et al., 2010).

Matrix-assisted laser desorption/ionization time of flight mass spectrometry (MALDI–TOF MS) promises a revolutionary breakthrough in clinical microbiology. The technology identifies bacteria within 6 min (Seng et al., 2009), with a species-level accuracy of 84.1–93.6% (Bizzini and Greub, 2010) and high sensitivity (~10^5^ CFU). Currently, MALDI-TOF MS has shown the ability to distinguish vanB-positive *Enterococcus faecium* from isolates that do not possess this resistance gene (Griffin et al., 2012) and demonstrate the carbapenemase activity by detection of meropenem and the relevant degradation products (Hrabak et al., 2011). Another study focused on a proteome analysis of ampicillin-resistant *Fusobacterium nucleatum* was reported (Al-Haroni et al., 2008). Moreover, MALDI-TOF MS fingerprinting has been used to distinguish the different expression levels of cell wall components between resistant isolates and sensitive isolates (Xu et al., 2006). Recently, RMI by MALDI–TOF MS has been adopted in various protocols, including the commercial Sepsityper kit (Bruker Daltonics, Bremen, Germany; Martiny et al., 2012; Hazelton et al., 2014; Idelevich et al., 2014; Martinez et al., 2014; Schieffer et al., 2014; Morgenthaler and Kostrzewa, 2015), serum separator tubes (Stevenson et al., 2010; Schubert et al., 2011) and in-house methods (Martiny et al., 2012). These techniques return the RMI from positive bottles within 30 min to a few hours. They also reliably identify species-level GN and GP bacteria (with accuracies of 90 and 76%, respectively) and yeasts (66%) (Morgenthaler and Kostrzewa, 2015). However, the Sepsityper kit is expensive, whereas the in-house method involves multi-step washing/centrifugation and yields relatively low RMI accuracy for GP bacteria and yeasts.

Rapid ASTs have been attempted in several studies. For instance, methicillin-resistant staphylococci and vancomycin-resistant enterococci have been detected in real-time PCR–melt curve analysis (Chan et al., 2015). MALDI–TOF MS can detect subtle differences in isogenic *Staphylococcus aureus*, which determine the organism's resistance to methicillin or teicoplanin (Majcherczyk et al., 2006), ampicillin susceptibility (Grundt et al., 2012), and carbapenemase (Hrabák et al., 2012). All of the above methods are efficient; however, molecular methods cannot identify the expression of resistance genes, and other methods are limited to one or a few specific antibiotic determination profiles.

To overcome these difficulties, we determined the RMI and RMAST by a combined MALDI–TOF MS/Vitek AST system. Bacterial cells were directly extracted from positive sterile body fluid cultures in serum separator tubes, and proliferated in brain heart infusion broth (BHI) for 2–4 h.

This study aims to evaluate the reliability and accuracy of this protocol in fast pathogen diagnosis without additional costs and efforts.

## Materials and methods

### Location

This study was conducted in Huashan Hospital (affiliated with Fudan University), located in the center of Shanghai, China. Huashan Hospital is one of the largest (1300 beds) comprehensive teaching hospitals in China, handling ~8000 admissions per day.

### Clinical samples

Sterile body fluids, including blood, cerebrospinal fluid (CSF), pleural fluid, ascitic fluid, pericardial effusion, joint cavity fluid, and vitreous fluid, were injected into blood culture bottles to improve the positive rate of clinical samples. From September 2014 to August 2015, we enrolled the first positive culture from each patient. Thus, we collected 485 non-duplicated positive cultures.

### Blood culture (BC) bottles and BC system

The BC bottles were BacT/Alert aerobic/SA and anaerobic/SN (bioMérieux, Marcy l'Etoile, France), and BACTEC Plus Aerobic/F, BACTEC Plus Anaerobic/F and BACTEC Mycosis-IC/F (Becton Dickinson, USA; Figure S1). All bottles were incubated in automated BC systems (BacT/Alert 3D, bioMérieux and BACTEC FX, Becton Dickinson; Figure S1) until they tested positive. Positive cultures were analyzed immediately after a positive alert during laboratory hours (8 a.m. to 5 p.m.). Cultures that became positive later than this period were stored in the BC systems and analyzed the next morning.

### Positive cultures processing for RMI analysis and RMAST

Based on the Gram staining results, 5 ml (sterile body fluid positive culture and blood positive culture caused by GN or polymicrobial) or 10 ml (blood positive culture caused by other bacteria) were drawn into one or two serum separator tubes (BD Vacutainer SSTII Advance, USA). Bacteria were pelleted by centrifugation at 4000 g for 10 min. The bacteria became aggregated at the surface of the polymeric gel, while the red-blood-cell component was sedimented beneath the gel layer. The supernatant was discarded and the bacterial pellet was gently resuspended in 1 ml sterile distilled water, without disrupting the gel layer. The suspension was transferred to a sterile Eppendorf tube, mixed thoroughly, and centrifuged at 16,000 g for 1 min. The supernatant was discarded and the washing/centrifugation steps were repeated once. The pellets were resuspended in 100 μl sterile distilled water. A portion of the resuspension was drawn and subjected to an ethanol/formic acid extraction procedure. The rest of the resuspension was prepared for 0.5 McFarland (McF) and subjected to RMAST as described for standard AST.

Prior to the ethanol/formic acid extraction, pellets of blood cultures caused by other bacteria (but not those caused by GN or polymicrobial) were resuspended in 1 ml BHI for enrichment. GP bacteria and yeasts were inoculated at 37°C with shaking at 200 rpm for 2 and 4 h, respectively. The enrichments were pelleted at 16,000 g for 1 min. After discarding the supernatant, the pellets were washed with 1 ml sterile distilled water, and re-centrifuged at 16,000 g for 1 min. Again, the supernatant was discarded.

**Figure 1:**
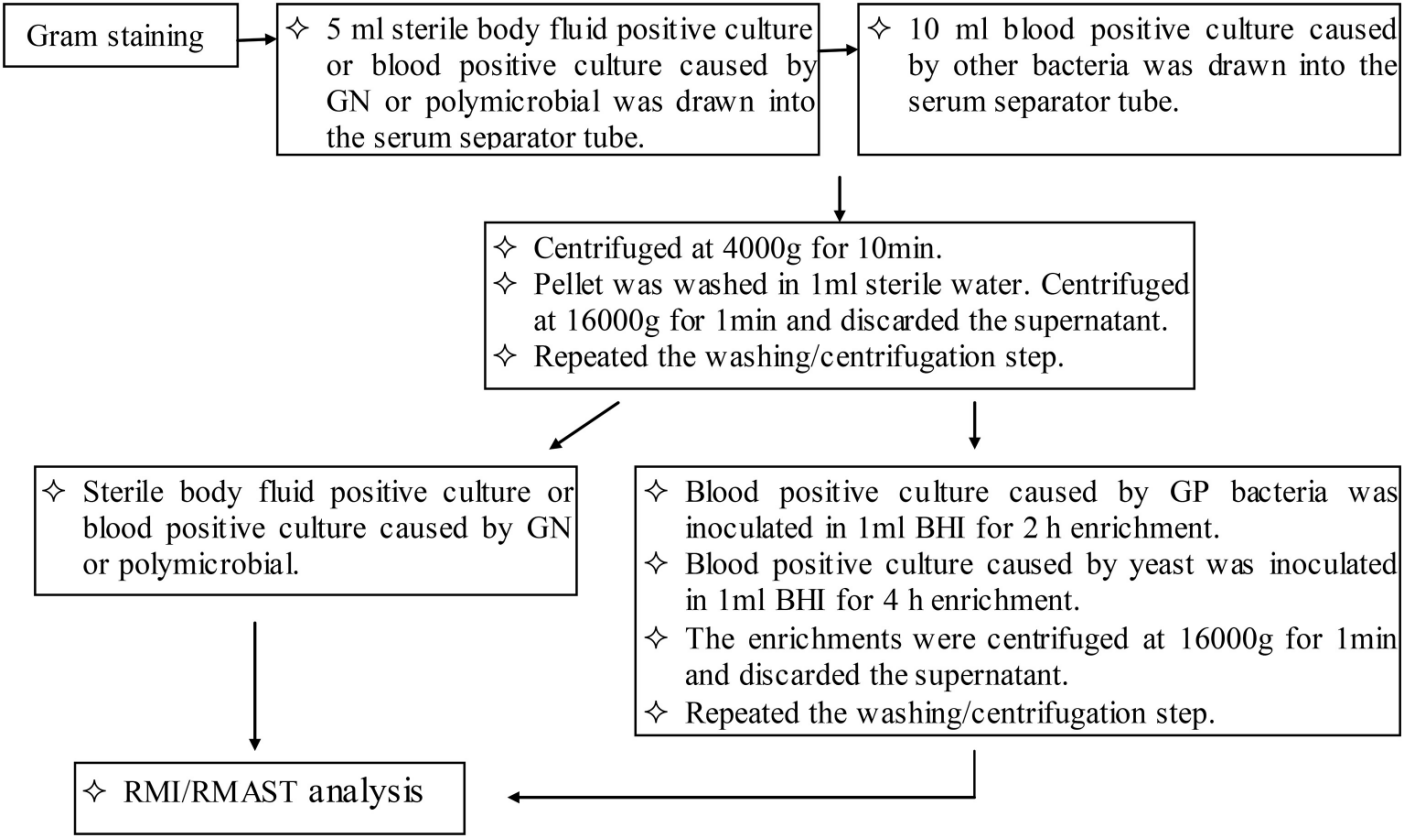
**Workflow of positive culture processing for RMI/RMAST analysis**.

In the ethanol/formic acid extraction procedure, a portion of the bacterial pellet was resuspended in 300 μl water by vortexing. The suspension was thoroughly mixed with 900 μl absolute ethanol and then centrifuged at 16,000 g for 1 min. The supernatant was discarded and the residual ethanol was removed after a repeat centrifugation. The cell pellet was air dried and dissolved in 30 μl of 70% formic acid by thorough vortexing. After adding 30 μl acetonitrile, the dissolved pellet was centrifuged at 16,000 g for 2 min and 1 μl supernatant was spotted onto a steel target plate for MALDI–TOF MS (Bruker Daltonics, Bremen, Germany) analysis (Figure 1).

### Standard identification and antimicrobial susceptibility testing

The positive broths were sub-cultured onto routing plates of 5% sheep blood agar, chocolate, or anaerobic blood agar. Bacteria that failed to grow on the routing plates were inoculated on self-made plates composed of 20 ml sterile blood culture broth (bioMérieux, Marcy l'Etoile, France; Becton Dickinson, USA) and 3.9% agar powder (Oxoid, Thermo Fisher Scientific, England). Plates were grown in the incubator (Thermo Scientific Forma, USA) at 35°C in 5% CO_2_ or an anaerobic atmosphere until visible colonies appeared. A pure bacterial colony was smeared onto a steel target plate for identification by MALDI–TOF MS. For yeasts, 1 μl of formic acid was added to the plate and air dried for 5 min.

In the antimicrobial susceptibility testing, Vitek cards AST-GN13, AST-Gp67, and AST-Gp68 were used for GN bacteria, staphylococci/enterococci and *Streptococcus pneumoniae*, respectively. Other streptococci and yeasts were tested with ATB-STREP5 and ATB-FUNGUS3 (bioMérieux, Marcyl' Etoile, France), respectively. The GN bacteria, staphylococci, enterococci, streptococci, or yeast pellets were dissolved in 0.45% saline solution to prepare 0.5–0.63 McF or 2 McF suspension, respectively. The Vitek card AST-GN13 or AST-Gp67/AST-Gp68 were filled with suspension composed of 3 ml 0.45% saline solution and 145 or 280 μl 0.5–0.63 McF suspension, respectively. The ATB-STREP5 strip was filled with suspension composed of ATB S medium and 200 μl 0.5 McF suspension. The ATB-FUNGUS3 strip was filled with suspension composed of ATB F2 medium and 20 μl 2McF suspension.

### Matrix-assisted laser desorption/ionization-time of flight mass spectrometry

**Figure 2:**
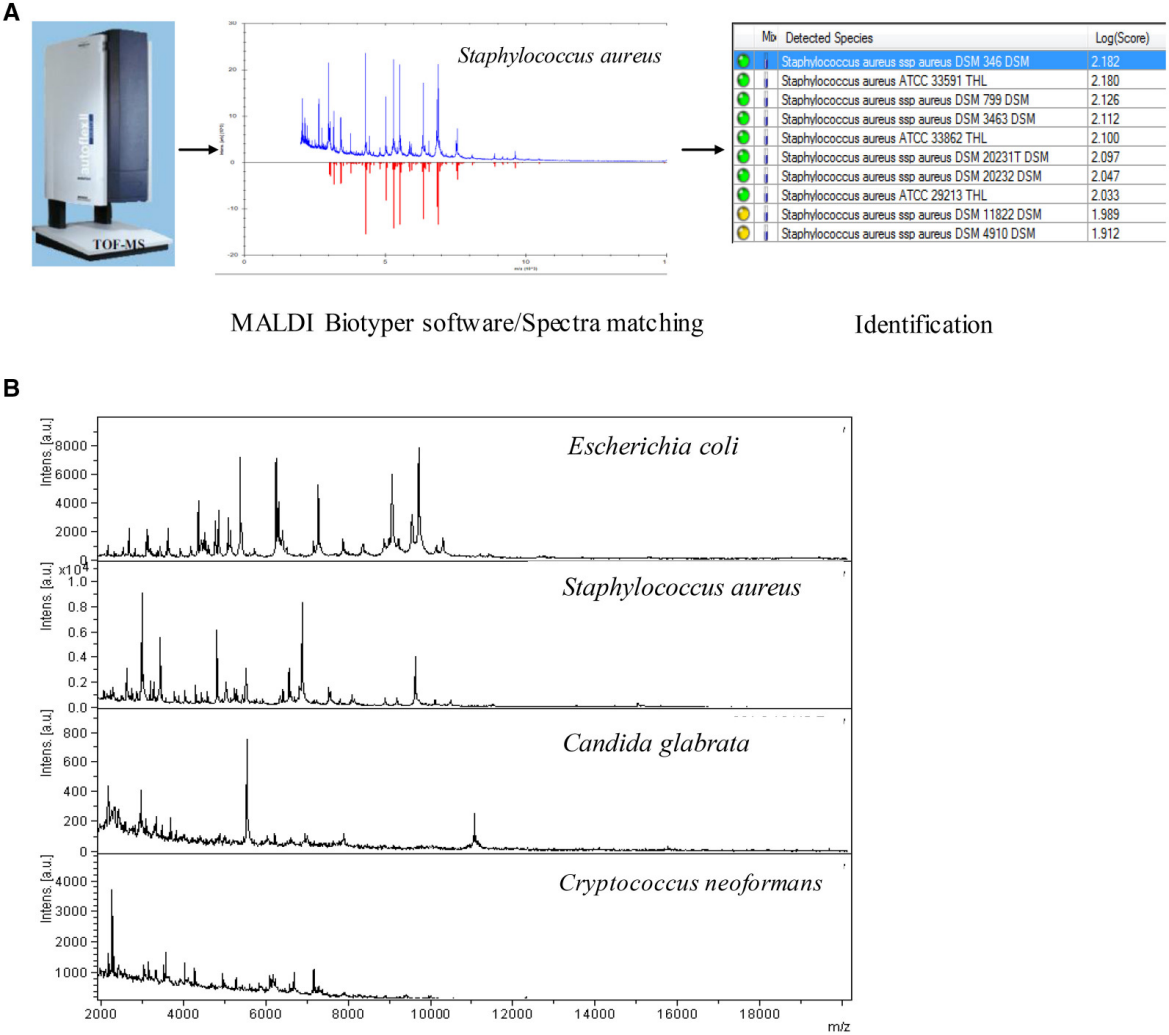
**(A)** The RMI workflow of MALDI-TOF MS system. **(B)** RMI spectra of representative bacterial species.

The spot was overlaid with 1 μl MALDI matrix (a saturated solution of α-cyano-4-hydroxycinnamic acid in 50% acetonitrile–2.5% trifluoroacetic acid). After drying at room temperature for 5 min, sample was subjected to analysis of the bacteria protein using MALDI-TOF MS system. The spectrum was obtained in linear positive-ion mode range from 2000 to 20 000 Da. Each spot was measured manually on five different positions by using 1000 laser shots at 25 Hz in groups of 40 shots. The MALDI Bruker Biotyper 3.0 software and library (Bruker Daltonics) were used for spectra analysis. According to the spectra matching and score criterion, MALDI Bruker Biotyper 3.0 software obtained appropriate scores and identification results. The workflow was shown in Figure 2A.

### Interpretation of RMI and RMAST

RMI results were scored by the manufacturer's standard criterion (cut-off values of 1.7 and 2.0 for acceptable identification to the genus and species levels, respectively) and by the modified criterion (cut-off values of 1.5 and 1.8 for at least three identical results in the list at the genus and species levels, respectively). These criteria have been described in previous studies (Schmidt et al., 2012; Machen et al., 2014; Martinez et al., 2014; Morgenthaler and Kostrzewa, 2015). The RMI results were compared with the final reports. Non-identifiable samples included correctly identified samples with scores below the genus level cut-off value and samples that presented no peak or a very weak signal. Discordant identification included samples that the RMI results were inconsistent with the final reports which proved correct. Discrepancies were resolved by 16S rRNA (for bacteria; primer F, 5′-AGAGTTTGATGATGGCTCAG-3′; primer R, 5′-ACCGCAACTGCTGGCAC-3′; expected PCR product size: ~800 bp) or 18S rRNA (for fungi; primer F, 5′-GATACCGTCGTAGTCTTA-3′; primer R, 5′-ATTCCTCGTTGAAGAGC-3′; expected PCR product size: ~800 bp) amplification. DNA was extracted from colonies sub-cultured for 24 h using Genomic DNA isolation kit (Sangon Biotech, Shanghai, China). Thermal cycler conditions were 94°C for 5 min, followed by 35 cycles of 94°C (30 s), 55°C (30 s), and 72°C (30 s), with a final extension at 72°C (7min).

RMAST results were compared with those obtained from the standard method. The minimum inhibitory concentrations (MIC) obtained by both methods were translated into clinical categories (susceptible, intermediate, resistant), following the CLSI recommendations. The comparison between the direct and standard inoculation methods was categorized as agreement, very major error (VME, false susceptibility), major error (ME, false resistance), or minor error (mE, susceptible/resistant versus intermediate susceptibility). Discrepancies in MICs were resolved by broth dilution methods according to the CLSI (2015) guidelines.

### Evaluation of clinical relevance

To prevent the development of resistance, to reduce toxicity, and to reduce costs, the antimicrobial regimen should be ceased when detected typical contaminants (Monoculture grew Coagulase-negative *staphylococcus* among multiple cultures, *Micrococcus* spp, *Corynebacterium* spp, *Bacillus* spp, *Propionibacterium acnes*) and assessed for potential de-escalation (De-escalation refers to narrow the spectrum of antimicrobial coverage and choose the most appropriate single-agent therapy). Antibiotic therapy should be installed when detected pathogenic species that cannot be distinguished by Gram staining and modified when detected intrinsic resistant bacteria (Bacteria were intrinsic antibiotic-resistance. *Stenotrophomonas maltophilia, Enterococcus casselifavus*, and *Candida glabrata* are intrinsic resistant to carbapenem, vancomycin, and fluconazole, respectively) or acquired resistant bacteria (Antibiotic-resistance was mediated by plasmid, resistant enzymes, or other resistance mechanisms). RMI/RMAST results were reported to the clinician. To assess the impact of our approach on the management of sterile body infections, we determined whether the results can instructively recommend an optimal therapeutic scheme. Unreliable results were excluded. Indications leading to a treatment change are listed in Table 1.

**Table 1:** **Indications for optimized antibiotic therapy and recommendations based on RMI /RMAST results**.

	**RMI result (0.58–4.58 h)**	**RMAST result (8.4–34 h)**
Cessation of antibiotic therapy	Detection of typical contaminants:	
	^*^ Monoculture grew Coagulase-negative *staphylococcus* among multiple cultures	
	^*^Micrococcus spp.	
	^*^Corynebacterium spp.	
	^*^Bacillus spp.	
	^*^Propionibacterium acnes	
Installation of antibiotic therapy	Detection of pathogenic species that cannot be distinguished by Gram staining:	Assessment and verification the initiated measure.
	^*^Staphylococcus aureus	
	^*^Staphylococcus lugdunensis	
	^*^Listeria monocytogenes	
Modification of antibiotic therapy	Detection of intrinsic resistant bacteria:	Assessment and verification the initiated measure.
	^*^Stenotrophomonas maltophilia with carbapenem resistance	
	^*^Enterococcus casselifavus with vancomycin resistance	
	^*^Candida glabrata with fluconazole resistance	
De-escalation antibiotic therapy		De-escalation of broad spectrum antibiotics or last-resort antibiotics (carbapenems, vancomycin, linezolid) or combination therapy in case of results indicates low-risk intrinsic resistant bacteria (e.g., *Escherichia coli, Staphylococci*) associated with negative acquired antibiotic-resistant history.
Modification of antibiotic therapy		Detection of acquired resistant bacteria (e.g., carbapenem resistant GN bacteria, vancomycin, or linezolid non-susceptible *Enterococci* and *Staphylococci*).

### Statistical analysis

Statistical time comparisons were conducted by a Wilcoxon signed-rank test (GraphPad Prism 5.0, CA, USA). *P* < 0.05 was considered statistically significant. All statistical tests were two-tailed.

## Results

### Rapid microorganism identification

Regardless of enrichment, the RMI test of each sample was completed in 40 min (15 min for the pellet collection/washing/centrifugation steps, 15 min for the extraction procedure, 5 min for the sample spotting/drying steps, 5 min for MALDI–TOF MS measurement). The RMI was evaluated in 485 non-duplicated positive cultures (Table 2). The compositions of the positive cultures are listed in Table S1.

**Table 2:** **RMI results scored by standard criterion and modified criterion**.

**Species**	**Standard cut-off values**				**Modified cut-off values**				**Not identified**	**Discordant ID**	**Mean scores (min–max)**
	**No ID *n* (%)**	**Correct ID**		**Not identified**	**Discordant ID**	**No ID *n* (%)**	**Correct ID**				
		**species Score ≥ 2.0**	**genus Score ≥ 1.7**				**species Score ≥ 1.8**	**genus Score ≥ 1.5**			
**AEROBIC GN BACTERIA**											
**Enterobacteriaceae**											
*Escherichia coli*	63 (100)	55 (87.3)	8 (12.7)	0	0	63 (100)	57 (90.5)	6 (9.5)	0	0	2.097 (1.712–2.417)
*Klebsiella pneumoniae*	38 (100)	33 (86.8)	5 (13.2)	0	0	38 (100)	37 (97.4)	1 (2.6)	0	0	2.076 (1.799–2.380)
*Klebsiella oxytoca*	1 (100)	1 (100)	0	0	0	1 (100)	1 (100)	0	0	0	2.261
*Enterobacter cloacae*	7 (100)	4 (57.1)	3 (42.9)	0	0	7 (100)	5 (71.4)	2 (28.6)	0	0	1.952 (1.762–2.087)
*Enterobacter aerogenes*	1 (100)	1 (100)	0	0	0	1 (100)	1 (100)	0	0	0	2.183
*Serratia marcescens*	4 (100)	2 (50.0)	2 (50.0)	0	0	4 (100)	2 (50.0)	2 (50.0)	0	0	1.928 (1.733–2.154)
*Morganella morganii*	3 (100)	3 (100)	0	0	0	3 (100)	3 (100)	0	0	0	2.217 (2.059–2.415)
*Proteus mirabilis*	3 (100)	2 (66.7)	1 (33.3)	0	0	3 (100)	3 (100)	0	0	0	2.079 (1.828–2.233)
*Proteus vulgaris*	2 (100)	1 (50.0)	1 (50.0)	0	0	2 (100)	1 (50.0)	1 (50.0)	0	0	1.901 (1.787–2.015)
*Salmonella*	2 (100)	0	2 (100)	0	0	2 (100)	1 (50.0)	1 (50.0)	0	0	1.752 (1.703–1.800)
*Pantoea*	2 (100)	1 (50.0)	1 (50.0)	0	0	2 (100)	2 (100)	0	0	0	2.079 (1.828–2.233)
*Raoultella ornithinolytica*	2 (100)	1 (50.0)	1 (50.0)	0	0	2 (100)	1 (50.0)	1 (50.0)	0	0	2.083 (1.794–2.372)
*Providencia rettgeri*	2 (100)	0	2 (100)	0	0	2 (100)	0	2 (100)	0	0	1.720 (1.712–1.727)
*Providencia stuartii*	1 (100)	0	1 (100)	0	0	1 (100)	1 (100)	0	0	0	1.890
*Citrobacter freundii*	2 (100)	2 (100)	0	0	0	2 (100)	2 (100)	0	0	0	2.044 (2.001–2.087)
*Citrobacter braakii*	1 (100)	0	1 (100)	0	0	1 (100)	1 (100)	0	0	0	1.924
Subtotal	134 (100)	106 (79.1)	28 (20.9)	0	0	134 (100)	118 (88.1)	16 (11.9)	0	0	
***ACINETOBACTER*** **SPP**											
*Acinetobacter baumannii*	21 (100)	17 (81.0)	4 (19.0)	0	0	21 (100)	18 (85.7)	3 (14.3)	0	0	2.054 (1.738–2.237)
*Acinetobacter junii*	3 (100)	2 (66.7)	1 (33.3)	0	0	3 (100)	2 (66.7)	1 (33.3)	0	0	1.947 (1.720–2.067)
*Acinetobacter haemolyticus*	3 (100)	0	3 (100)	0	0	3 (100)	2 (66.7)	1 (33.3)	0	0	1.755 (1.702–1.817)
*Acinetobacter pittii*	1 (100)	0	1 (100)	0	0	1 (100)	1 (100)	0	0	0	1.826
Subtotal	28 (100)	19 (67.9)	9 (32.1)	0	0	28 (100)	23 (82.1)	5 (17.9)	0	0	
**NON-FERMENTERS**											
*Pseudomonas aeruginosa*	12 (100)	8 (66.7)	4 (33.3)	0	0	12 (100)	10 (83.3)	2 (16.7)	0	0	1.999 (1.701–2.214)
*Stenotrophomonas maltophilia*	5 (20.0)	0	1 (20.0)	4 (80.0)	0	5 (20.0)	3 (60.0)	1 (20.0)	1 (20.0)	0	N/A
*Pseudomonas fulva*	1 (100)	0	1 (100)	0	0	1 (100)	1 (100)	0	0	0	1.994
*Myrodies odoratimus*	1 (100)	1 (100)	0	0	0	1 (100)	1 (100)	0	0	0	2.206
*Pseudomonas oryzihabitans*	1 (100)	0	1 (100)	0	0	1 (100)	1 (100)	0	0	0	1.878
*Brevendimonas diminuta*	1 (0)	0	0	1 (100)	0	1 (0)	0	0	1 (100)	0	N/A
*Aeromona hydrophila*	2 (100)	1 (50.0)	1 (50.0)	0	0	2 (100)	2 (100)	0	0	0	1.986 (1.927–2.045)
*Aeromonas caviae*	1 (0)	0	0	1 (100)	0	1 (100)	0	1 (100)	0	0	1.682
*Aeromonas veronii*	1 (100)	0	1 (100)	0	0	1 (100)	0	1 (100)	0	0	1.765
*Methylobacterium*	1 (0)	0	0	1 (100)	0	1 (100)	0	1 (100)	0	0	1.623
*Vibrio cholerae*	1 (100)	0	1 (100)	0	0	1 (100)	1 (100)	0	0	0	1.876
Subtotal	27 (74.0)	10 (37.0)	10 (37.0)	7 (26.0)	0	27 (92.6)	19 (70.4)	6 (22.2)	2 (7.4)	0	
Total	189 (96.3)	135 (71.4)	47 (24.9)	7 (3.7)	0	189 (98.9)	160 (84.7)	27 (16.9)	2 (1.1)	0	
**AEROBIC GP BACTERIA**											
**Staphylococci**											
*Staphylococcus aureus*	41 (100)	19 (46.3)	22 (53.7)	0	0	41 (100)	31 (75.6)	10 (24.4)	0	0	1.989 (1.700–2.410)
*Staphylococcus epidermidis*	29 (82.8)	6 (20.7)	18 (62.1)	3 (10.3)	2 (6.9)	29 (86.2)	14 (48.3)	11 (37.9)	2 (6.9)	2 (6.9)	1.850 (1.658–2.098)
*Staphylococcus capitis*	28 (85.7)	6 (21.4)	18 (64.3)	2 (6.9)	2 (6.9)	28 (89.3)	12 (42.6)	13 (46.4)	1 (3.8)	2 (6.9)	1.865 (1.616–2.159)
*Staphylococcus hominis*	23 (82.6)	4 (17.4)	15 (65.2)	2 (8.7)	2 (8.7)	23 (82.6)	9 (39.1)	10 (43.5)	2 (8.7)	2 (8.7)	1.857 (1.703–2.195)
*Staphylococcus haemolyticus*	9 (88.9)	0	8 (88.9)	1 (11.1)	0	9 (88.9)	0	8 (88.9)	1 (11.1)	0	1.748 (1.701–1.882)
*Staphylococcus cohnii*	3 (66.7)	0	2 (66.7)	1 (33.3)	0	3 (66.7)	0	2 (66.7)	1 (33.3)	0	1.769 (1.741–1.797)
*Staphylococcus warneri*	1 (100)	0	1 (100)	0	0	1 (100)	1 (100)	0	0	0	1.934
*Staphylococcus xylosus*	1 (100)	1 (100)	0	0	0	1 (100)	1 (100)	0	0	0	2.068
*Staphylococcus saprophyticus*	1 (100)	0	1 (100)	0	0	1 (100)	1 (100)	0	0	0	1.920
*Staphylococcus auricularis*	1 (100)	0	1 (100)	0	0	1 (100)	1 (100)	0	0	0	1.912
*Staphylococcus simulans*	1 (100)	0	1 (100)	0	0	1 (100)	1 (100)	0	0	0	1.819
*Staphylococcus caprae*	1 (0)	0	0	1 (100)	0	1 (100)	0	0	1 (100)	0	N/A
Subtotal	139 (88.5)	36 (25.9)	87 (62.6)	10 (7.2)	6 (4.3)	139 (89.9)	71 (51.1)	54 (38.8)	8 (5.8)	6 (4.3)	
**ENTEROCOCCI**											
*Enterococcus faecium*	13 (84.6)	2 (15.4)	9 (69.2)	1 (7.7)	1 (7.7)	13 (92.3)	8 (61.5)	4 (30.8)	0	1 (7.7)	1.856 (1.606–2.113)
*Enterococcus faecalis*	10 (100)	4 (40.0)	6 (60.0)	0		10 (100)	8 (80.0)	2 (20.0)	0	0	1.926 (1.742–2.186)
*Enterococcus casselifavus*	4 (100)	3 (75.0)	1 (25.0)	0		4 (100)	3 (75.0)	1 (25.0)	0	0	1.989 (1.710–2.097)
*Enterococcus avium*	3 (66.7)	0	2 (66.7)	1 (33.3)		3 (66.7)	0	2 (66.7)	1 (33.3)	0	1.778 (1.760–1.795)
Subtotal	30 (90.0)	9 (30.0)	18 (60.0)	2 (6.7)	1 (3.3)	30 (93.3)	19 (63.3)	9 (30.0)	1 (3.3)	1 (3.3)	
**STREPTOCOCCUS**											
*Streptococcus pneumoniae*	5 (40.0)	0	2 (40.0)	2 (40.0)	1 (20.0)	5 (60.0)	2 (40.0)	1 (20.0)	1 (20.0)	1 (20.0)	N/A
*Streptococcus agalactiae*	3 (100)	0	3 (100)	0	0	3 (100)	3 (100)	0	0	0	1.898 (1.870–1.934)
*Streptococcus pyogenes*	1 (0)	0	0	1 (100)	0	1 (0)	0	0	1 (100)	0	N/A
*Streptococcus salivarius*	5 (60.0)	1 (20.0)	2 (40.0)	2 (40)	0	5 (80.0)	1 (20)	3 (60.0)	1 (20)	0	1.862 (1.726–2.088)
*Streptococcus sanguis*	2 (0)	0	0	2 (100)	0	2 (50.0)	0	1 (50.0)	1 (50)	0	N/A
*Streptococcus mitis*	2 (100)	0	2 (100)	0	0	2 (100)	0	2 (100)	0	0	1.729 (1.702–1.755)
*Streptococcus gordonii*	1 (100)	0	1 (100)	0	0	1 (100)	0	1 (100)	0	0	1.721
*Streptococcus oralis*	1 (0)	0	0	1 (100)	0	1 (100)	0	1 (100)	0	0	N/A
*Streptococcus massiliensis*	1 (0)	0	0	1 (100)	0	1 (0)	0	0	1 (100)	0	N/A
Subtotal	21 (52.4)	1 (4.8)	10 (47.6)	9 (42.9)	1 (4.8)	21 (71.4)	6 (28.6)	9 (42.9)	5 (23.8)	1 (4.8)	
**OTHER GP BACTERIA**											
*Listeria monocytogenes*	4 (100)	4 (100)	0	0	0	4 (100)	4 (100)	0	0	0	2.125 (2.078–2.168)
*Micrococcus spp*	2 (50.0)	0	1 (50.0)	0	1 (50.0)	2 (50.0)	0	1 (50.0)	0	1 (50.0)	1.773
*Corynebacterium spp*	6 (16.7)	0	1 (16.7)	5 (83.3)	0	6 (50.0)	0	3 (50.0)	3 (50.0)	0	N/A
*Bacillus amyloliquefaciens*	1 (100)	1 (100)	0	0	0	1 (100)	1 (100)	0	0	0	2.083
Subtotal	13 (53.8)	5 (38.5)	2 (15.4)	5 (38.5)	1 (7.7)	13 (69.2)	5 (38.5)	4 (30.8)	3 (23.1)	1 (7.7)	
Total	203 (82.8)	51 (25.1)	117 (57.6)	26 (12.8)	9 (4.4)	203 (87.2)	101 (49.8)	76 (37.4)	17 (8.4)	9 (4.4)	
**FASTIDIOUS BACTERIA**											
*Haemophilus influenzae*	2 (50.0)	0	1 (50.0)	1 (50.0)	0	2 (50.0)	1 (50.0)	0	1 (50.0)	0	1.888
*Erysipelothrix rhusiopathiae*	1 (100)	0	1 (100)	0	0	1 (100)	0	1 (100)	0	0	1.795
*Actinobacillus actinomycetemcomitans*	1 (0)	0	0	1 (100)	0	1 (100)	0	0	1 (100)	0	N/A
*Actinomyces neuii*	1 (0)	0	0	1 (100)	0	1 (0)	0	0	1 (100)	0	N/A
*Actinomyces meyeri*	1 (0)	0	0	1 (100)	0	1 (0)	0	0	1 (100)	0	N/A
*Nocardia* spp	4 (0)	0	0	4 (100)	0	4 (0)	0	0	4 (100)	0	N/A
*Streptomyces*	1 (0)	0	0	1 (100)	0	1 (0)	0	0	1 (100)	0	N/A
*Granulicatella adiacens*	2 (0)	0	0	2 (100)	0	2 (0)	0	0	2 (100)	0	N/A
*Moraxella* spp	1 (0)	0	0	1 (100)	0	1 (0)	0	0	1 (100)	0	N/A
*Cardiobacterium hominis*	1 (0)	0	0	1 (100)	0	1 (0)	0	0	1 (100)	0	N/A
*Lactobacillus*	1 (0)	0	0	1 (100)	0	1 (0)	0	0	1 (100)	0	N/A
*Microbacterium liquefaciens*	1 (0)	0	0	1 (100)	0	1 (0)	0	0	1 (100)	0	N/A
Total	17 (11.8)	0 (0.0)	2 (11.8)	15 (88.2)	0	17 (11.8)	1 (5.9)	1 (5.9)	15 (88.2)	0	
**FUNGI**											
*Candida albicans*	7 (42.9)	2 (28.6)	1 (14.3)	3 (42.8)	1 (14.3)	7 (71.4)	3 (42.9)	2 (28.6)	2 (14.3)	1 (14.3)	N/A
*Candida tropicalis*	6 (50.0)	0	3 (50.0)	2 (33.3)	1 (16.7)	6 (83.3)	3 (50.0)	2 (33.3)	0	1 (16.7)	1.808 (1.696–1.884)
*Candida glabrata*	3 (33.3)	0	1 (33.3)	1 (33.3)	1 (33.3)	3 (33.3)	1 (33.3)	0	1 (33.3)	1 (33.3)	N/A
*Candida parapsilosis*	3 (33.3)	0	1 (33.3)	2 (66.7)	0	3 (66.7)	1 (33.3)	1 (33.3)	1 (33.3)	0	N/A
*Candida lusitaniae*	1 (100)	0	1 (100)	0	0	1 (100)	0	1 (100)	0	0	1.782
*Cryptococcus neoformans*	15 (86.7)	9 (60.0)	4 (26.7)	1 (6.7)	1 (6.7)	15 (86.7)	12 (80.0)	1 (6.7)	1 (6.7)	1 (6.7)	1.928 (1.626–2.237)
*Cryptococcus albidus*	1 (0)	0	0	1 (100)	0	1 (0)	0	0	1 (100)	0	N/A
*Trichosporon asahii*	1 (100)	1 (100)	0	0	0	1 (100)	1 (100)	0	0	0	2.029
Total	37 (62.2)	12 (32.5)	11 (29.7)	10 (27.0)	4 (10.8)	37 (75.7)	21 (56.8)	7 (18.9)	5 (13.5)	4 (10.8)	
**ANAEROBIC BACTERIA**											
*Bacteroides fragilis*	9 (100)	5 (55.6)	4 (44.4)	0	0	9 (100)	9 (100)	0	0	0	2.016 (1.820–2.283)
*Propionibacterium acnes*	3 (100)	2 (66.7)	1 (33.3)	0	0	3 (100)	2 (66.7)	1 (33.3)	0	0	2.020 (1.736–2.217)
*Fusobacterium necrophorum*	2 (50.0)	0	1 (50.0)	1 (50.0)	0	2 (50.0)	0	1 (50.0)	1 (50.0)	0	1.798
*Bacteroides uniformis*	1 (100)	1 (100)	0	0	0	1 (100)	1 (100)	0	0	0	2.008
*Veillonella*	1 (100)	1 (100)	0	0	0	1 (100)	1 (100)	0	0	0	2.038
*Staphylococcus saccharolylicus*	1 (100)	0	1 (100)	0	0	1 (100)	1 (100)	0	0	0	1.828
*Prevotella bivia*	1 (100)	0	1 (100)	0	0	1 (100)	1 (100)	0	0	0	1.894
*Campylobacter fetus*	1 (0)	0	0	1 (100)	0	1 (100)	0	1 (100)	0	0	N/A
Total	19 (89.5)	9 (47.4)	8 (42.1)	2 (10.5)	0	19 (94.7)	15 (78.9)	3 (15.8)	1 (5.3)	0	
Polymicrobial	16 (81.2)	5 (31.3)	8 (50.0)	3 (18.8)	0	16 (81.2)	7 (43.8)	6 (37.5)	3 (18.8)	0	1.924 (1.703-2.241)
**BACTERIA GROW IN THE SPECIAL MEDIUM**											
*Methylobacterium*	1 (100)	0	0	1 (100)	0	1 (100)	0	1 (100)	0	0	1.588
*Streptococcus salivarius thermophilus*	1 (100)	0	1 (100)	0	0	1 (100)	1 (100)	0	0	0	1.573
*Staphylococcus saccharolylicus*	1 (100)	0	1 (100)	0	0	1 (100)	1 (100)	0	0	0	1.828
*Campylobacter fetus*	1 (0)	0	0	1 (100)	0	1 (100)	0	1 (100)	0	0	N/A
Total	4 (50.0)	0 (0.0)	2 (50.0)	2 (50.0)	0	4 (100)	2 (50.0)	2 (50.0)	0 (0.0)	0	

*FID, final identification; FAST, final AST; N/A, not available*.

The RMI spectra of representative bacterial species were shown in Figure 2B. Scored by the standard criterion, the correct identification rates of RMI were 96.3% for GN bacteria (182/189), 82.8% for GP bacteria (168/203), 11.8% for fastidious bacteria (2/17), 62.2% for fungi (23/37), and 89.5% for anaerobic bacteria (17/19). In cultures of polymicrobial, the dominant bacterium was correctly identified in 81.2% (13/16) of cases and bacteria that failed to grow on the routing plates were identified with a success rate of 50.0% (2/4). Scored by the modified criterion, the correct identification rates of RMI were 98.9% (187/189) for GN bacteria, 87.2% (177/203) for GP bacteria, 11.8% (2/17) for fastidious bacteria, 75.7% (28/37) for fungi, and 94.7% (18/19) for anaerobic bacteria (18/19). In cultures containing polymicrobial and bacteria that failed to grow on the routing plates, the correct identification rates were 81.2% (13/16; dominant species) and 100.0% (4/4), respectively. The modified criterion provided more accurate RMI scores than the standard criterion, with no relevant misidentification at the genus level (Tables 2, 3). The RMI accuracy was lower in blood samples than in other sterile body fluids (Table S1). The mean RMI scores and the RMI results of polymicrobial are listed in Tables 2, 3, respectively.

**Table 3:** **RMI results of polymicrobial**.

**Species**	**Correct ID**	**Scores**
*Stenotrophomonas maltophilia/Staphylococcus haemolyticus*	*Stenotrophomonas maltophilia*	1.732
*Klebsiella pneumoniae/Enterococcus faecalis*	*Klebsiella pneumonia Enterococcus faecalis*	2.316/2.482
*Enterobacter cloacae/Streptococcus oralis*	*Not identified (very weak signal)*	N/A
*Candida tropicalis/Acinetobacter baumannii*	*Acinetobacter baumannii*	1.703
*Streptococcus oralis/Staphylococcus epidermidis*	*Not identified (Misidentified for Staphylococcus warneri)*	1.752
*Acinetobacter baumannii/Klebsiella pneumoniae*	*Klebsiella pneumoniae*	2.176
*Staphylococcus capitis/Klebsiella pneumoniae*	*Staphylococcus capitis*	1.739
*Pseudomonas aeruginosa/Klebsiella pneumoniae*	*Klebsiella pneumoniae*	1.824
*Pseudomonas aeruginosa/Klebsiella pneumoniae*	*Klebsiella pneumoniae*	2.219
*Pseudomonas aeruginosa/Klebsiella pneumoniae*	*Klebsiella pneumoniae*	2.22
*Staphylococcus epidermidis/Streptococcus salivarius*	*Staphylococcus epidermidis*	1.902
*Enterococcus faecium/Staphylococcus haemolyticus*	*Not identified (very weak signal)*	N/A
*Staphylococcus haemolyticus/Staphylococcus capitis*	*Staphylococcus capitis*	1.749
*Acinetobacter baumannii/Klebsiella pneumoniae*	*Klebsiella pneumoniae*	2.241
*Staphylococcus aureus/Pantoea spp*	*Staphylococcus aureus*	1.715
*Acinetobacter baumannii/Klebsiella pneumoniae*	*Klebsiella pneumoniae*	2.241

### Rapid antimicrobial susceptibility test

Among the 320 non-duplicated positive cultures, 140 GN bacteria, 105 *Staphylococcus* spp., 28 *Enterococcus* spp., 14 *Streptococcus* spp., 33 fungi, and 3349 bacterial /antimicrobial combinations, were analyzed in the RMAST investigation (Table 4).

**Table 4:** **Bacterial/antimicrobial combinations and errors in bacterial isolates from positive-culture sterile body fluids**.

**Antimicrobial agent**	**No**	**Category agreement**		**mE**		**ME**		**VME**	
**GN BACTERIA (***n* = 140**)**									
Amikacin	122	120	98.36%	1	0.82%			1	0.82%
Gentamicin	135	131	97.04%	3	2.22%			1	0.74%
Ciprofloxacin	135	131	97.04%	3	2.22%	1	0.74%		
Ampicillin	117	117	100.0%						
Cefazolin	135	127	94.07%	7	5.19%			1	0.74%
Cefotetan	135	129	95.56%	4	2.96%			2	1.48%
Ceftazidime	135	127	94.07%	6	4.44%	2	1.48%		
Cefotaxime	135	134	99.26%	1	0.74%				
Cefepime	135	123	91.11%	9	6.67%			3	2.22%
Imipenem	135	133	98.52%	2	1.48%				
Ertapenem	112	112	100.0%						
Piperacillin-tazobactam	122	116	95.08%	5	4.10%			1	0.82%
Sulfamethoxazole/trimethoprim	140	137	97.86%	2	1.43%	1	0.71%		
Tobramycin	135	132	97.78%	3	2.22%				
Total	1828	1769	96.77%	46	2.52%	4	0.22%	9	0.49%
***STAPHYLOCOCCUS*** **SPP (*n* = 105)**, ***ENTEROCOCCUS*** **SPP (*n* = 28)**									
Penicillin	105	104	99.05%					1	0.95%
Oxacillin	105	104	99.05%					1	0.95%
Gentamicin	105	83	79.05%	10	9.52%	5	4.76%	7	6.67%
Levofloxacin	133	124	93.23%	6	4.51%			3	2.26%
Erythromycin	133	130	97.74%	2	1.50%			1	0.75%
Clindamycin	133	127	95.49%	4	3.01%			2	1.50%
Linezolid	133	133	100.0%						
Vancomycin	133	133	100.0%						
Rifampicin	105	105	100.0%						
Sulfamethoxazole/trimethoprim	105	93	88.57%	1	0.95%	2	1.90%	9	8.57%
Ampicillin	28	4	14.29%	23	82.14%			1	3.57%
Gentamicin-High level	28	25	89.29%			2	7.14%	1	3.57%
Total	1246	1165	93.50%	46	3.69%	9	0.72%	26	2.09%
***STREPTOCOCCUS*** **SPP (*n* = 14)**									
Penicillin	14	13	92.86%			1	7.14%		
Cefotaxime	14	14	100.0%						
Ceftriaxone	5	5	100.0%						
Imipenem	5	5	100.0%						
Meropenem	5	5	100.0%						
Levofloxacin	14	14	100.0%						
Erythromycin	14	14	100.0%						
Clindamycin	14	14	100.0%						
Linezolid	5	5	100.0%						
Vancomycin	14	14	100.0%						
Quinuppistin-Dalfo	9	8	88.89%					1	11.11%
Tetracycline	9	9	100.0%						
Chloramphenicol	9	9	100.0%						
Sulfamethoxazole/trimethoprim	9	9	100.0%						
Total	140	138	98.57%			1	0.71%	1	0.71%
**YEAST (*n* = 18)**									
5-Fluorocytosine	18	17	94.44%	1	5.56%				
Fluconazole	18	17	94.44%					1	5.56%
Voriconazole	18	18	100.0%						
Amphotericin B	18	18	100.0%						
Itraconazole	18	16	88.89%	2	11.11%				
Total	90	86	95.56%	3	3.33%			1	1.11%
***CRYPTOCOCCUS NEOFORMANS*** **(*n* = 15)**									
5-Fluorocytosine	15	15	100.0%						
Fluconazole	15	15	100.0%						
Amphotericin B	15	15	100.0%						
Total	45	45	100.0%						

*The total numbers of isolates differed for each specific antimicrobial agent tested according to the CLSI (2015) guidelines*.

The RMAST was actually performed on 142 GN bacteria (109 Enterobacteriaceae, 14 *Acinetobacter* spp., and 19 non-fermentative bacteria), but two cultures (1 *Acinetobacter junii*, 1 *Brevundimonas diminuta*) grew poorly so were excluded from the analysis. In total, 1828 bacterial/antimicrobial combinations were analyzed with a category agreement of 96.77%, a mE rate of 2.52%, an ME rate of 0.22%, and a VME rate of 0.49%. Errors were found in *Escherichia coli* (1.09% mE, 0.05% ME, 0.27% VME), *Klebsiella pneumoniae* (0.66% mE, 0.05% ME, 0.71% VME), *Enterobacter cloacae* (0.22% mE), *Enterobacter aerogenes* (0.05% mE), *Morganella morganii* (0.16% mE), *Raoultella ornithinolytica* (0.11% mE), *Providencia rettgeri* (0.05% mE), *Pseudomonas aeruginosa* (0.22% mE, 0.05% ME, 0.11% VME), and other non-fermenters (0.22% mE, 0.11% ME, 0.05% VME).

Although, RMAST results were available for all 28 *Enterococcus* spp., they were available for only 105 of the 114 *Staphylococcus* spp.; 10 cases failed because of poor growth (4 *Staphylococcus epidermidis*, 2 *S. hominis*, 1 *S. capitis*, 1 *S. auricularis*, 1 *S. simulans*, and 1 *S. caprae*). In total, 1246 bacterial/antimicrobial combinations were analyzed with a category agreement of 93.50%, an mE rate of 3.69%, an ME rate of 0.72%, and a VME rate of 2.09%. Errors were found in *S. aureus* (0.32% mE, 0.16% VME), Coagulase-negative *staphylococcus* (1.28% mE, 0.56% ME, 1.76% VME), and *Enterococci* spp. (0.32% mE, 0.16% ME, 0.16% VME).

RMAST was originally performed on 21 *Streptococcus* spp., including 5 *S. pneumoniae*, 3 *S. agalactiae*, 1 *S. pyogenes*, and 12 *S. viridians*, but 7 of the *S. viridans* failed because of poor growth, yielding 14 RMAST results. In total, 140 bacterial/antimicrobial combinations were analyzed with a category agreement of 98.57%, an ME rate of 0.71%, and a VME rate of 0.71%.

RMAST results were available for 33 of the 37 fungal isolates (7 *Candida albicans*, 6 *C. tropicalis*, 3 *C. parapsilosis*, 3 *C. glabrata*, 1 *C. lusitaniae*, 1 Trichosporon asahii, 15 *Cryptococcus neoformans*, and 1 *C. albidus*). Four cases (1 *C. albicans*, 1 *C. parapsilosis, 1 C. glabrata*, and 1 Cryptococcus albidus) grew poorly and were thus excluded. In total, 90 bacterial/antimicrobial combinations were analyzed with a category agreement of 95.56%, an mE rate of 3.33% and a VME rate of 1.11% in the yeast group. Forty-five bacterial/antimicrobial combinations were also analyzed with a category agreement of 100% in the *C. neoformans* group.

Forty-three multi-drug resistant bacteria were detected in this study, including 1 vancomycin-intermediate *Staphylococcus epidermidis*, 1 vancomycin-resistant *Enterococcus faecium*, 1 linezolid-resistant *S. aureus*, 8 linezolid-resistant *S. capitis*, 16 carbapenem non-susceptible Enterobacteriaceae, 8 carbapenem non-susceptible *Acinetobacter* spp., and 6 carbapenem non-susceptible non-fermenting spp. The RMAST category agreement of multi-drug resistant bacteria was 97.68%, with 1 mE in carbapenem non-susceptible Enterobacteriaceae.

### Time to identification and antimicrobial susceptibility testing

**Figure 3:**
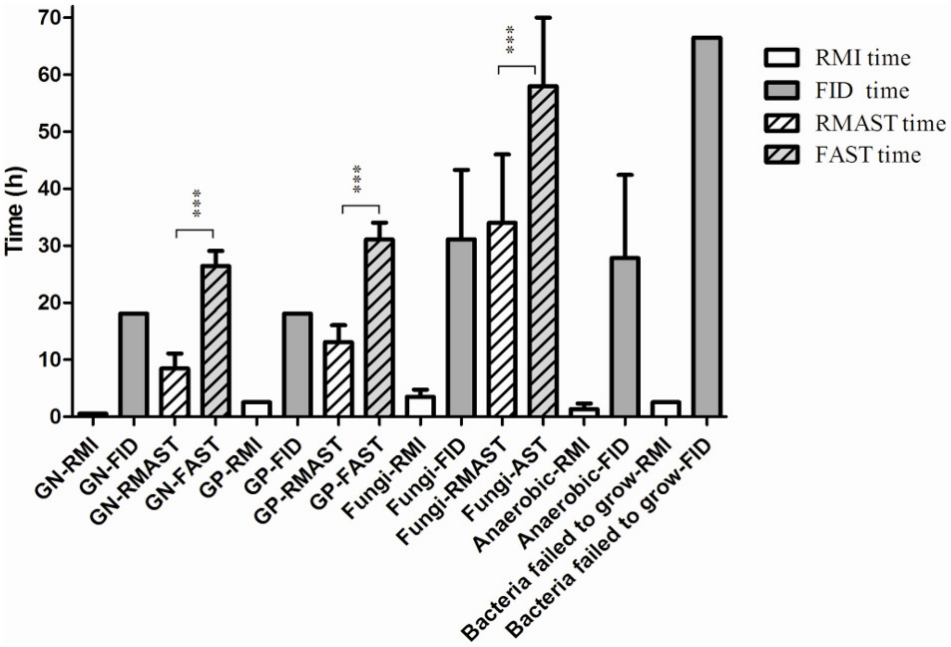
**Comparison between the average times of RMI/RMAST and FID/FAST**. The average times of RMAST vs. the conventional method were of great statistical significant (*p* < 0.0001). ^***^p < 0.0001. Bacteria failed to grow: bacteria failed to grow on the routing plates.

The average times of the RMI (vs. final identification report) were 0.58 h for GN, polymicrobial and sterile body fluid bacteria (vs. 18.1 h), 2.58 h for GP bacteria (vs. 18.1 h), 3.53 h for fungi (0.58 h in sterile body fluid and 4.58 h in blood, vs. 32.2 h), 1.36 h for anaerobic bacteria (0.58 h for GN and 2.58 h for GP bacteria, vs. 27.8 h), and 2.5 h for bacteria that failed to grow on the routing plates (vs. 64 h). The results are presented in Figure 3.

The average times of RMAST (vs. the conventional method) were 8.4 ± 2.6 h for GN bacteria (vs. 26.4 ± 2.6 h; *p* < 0.0001), 13.1 ± 3.0 h for GP bacteria (vs. 31.1 ± 3.0 h; *p* < 0.0001), 34 ± 12 h for fungi (vs. 58 ± 12 h; *p* < 0.0001; Figure 3).

### Analysis of clinical relevance

**Figure 4:**
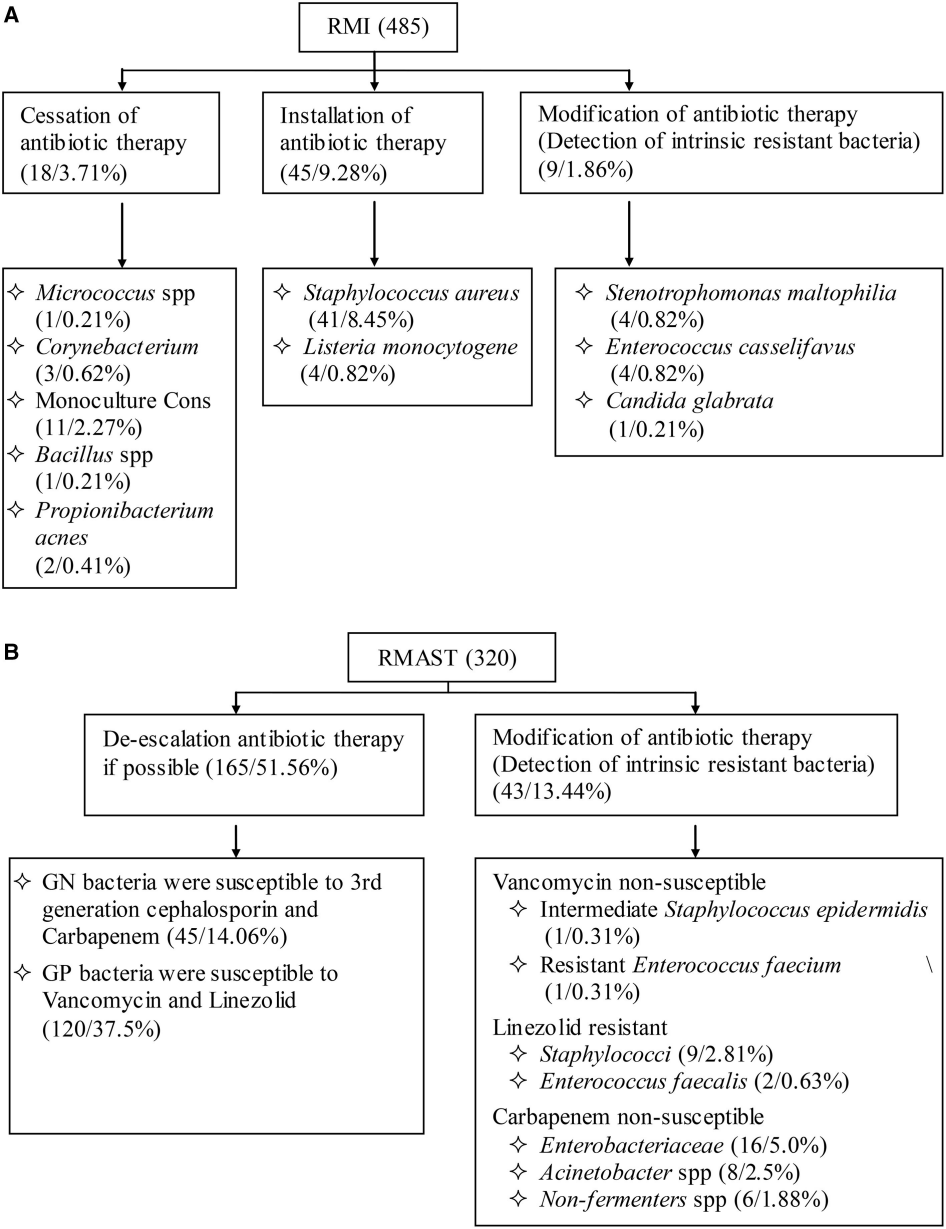
**(A):** Influence of RMI results on empirical antimicrobial therapy. **(B)**: Influence of RMAST results on empirical antimicrobial therapy.

Among 485 RMI cases (Figure 4), 14.85% of the results indicated that clinicians should adjust their treatment. Specifically, antibiotic therapy should be ceased in 3.71% of patients, initiated in 9.28% of patients, and modified in 1.86% of patients. Among the 320 RMAST results (Figure 4), 65% indicated that clinicians could optimize the therapeutic regimen by de-escalating (51.6%) or modifying (13.44%) the antibiotic therapy.

## Discussion

Sterile body fluid infections tend to trigger systemic or local tissue damage. Because of their severity and morbidity, BSIs and acute bacterial meningitis have placed a great burden on health care settings (Adhikari et al., 2010; Thigpen et al., 2011; Goto and Al-Hasan, 2013; Portnoy et al., 2015). Early diagnosis and rapid intervention are critical to improving patient prognosis.

This study incorporated a combined MALDI–TOF MS/Vitek AST system into the clinical microbiology laboratory workflow. The aim was to provide clinicians with fast and accurate identification results and multiple precise MIC values for optimizing the therapeutic regimen.

Numerous strategies can identify RMI pathogens from positive blood samples by MALDI–TOF MS (Hazelton et al., 2014; Idelevich et al., 2014; Martinez et al., 2014; Schieffer et al., 2014; Chan et al., 2015; Morgenthaler and Kostrzewa, 2015). The RMI accuracy of GN and GP bacteria or yeasts identified by these strategies has been widely reported, but the investigated species were mostly single. Comprehensive analyses are relatively rare. In this study, we collected 485 non-duplicated positive cultures from blood, csf, ascitic fluid, vitreous fluid, and eye tissues samples, and initiated by GN and GP bacteria, streptococci, fungi, fastidious bacteria, anaerobic bacteria, polymicrobial, and bacteria that failed to grow on the routing plates, and systematically evaluated the accuracy and feasibility of RMI. Data showed the RMI accuracies of CSF, ascitic fluid, vitreous fluid, and eye tissues (almost 100%) were obviously higher than blood sample (57.1–100%), especially for CSF, ascitic fluid, vitreous fluid, and eye tissues samples which might attribute to the blood protein interference. Moreover, the correct RMI rate of GN and GP bacteria reached 98.9 and 87.2%, respectively, similar to the results of previous studies (Hazelton et al., 2014; Schieffer et al., 2014), whereas that of fungi (75.7%) was slightly higher than in previous studies (Idelevich et al., 2014). We attribute this improvement to the 4 h enrichment and constitution of the sample source. The RMI accuracy of csf infections caused by *C. neoformans* was especially high (100%, *n* = 9). Fastidious bacteria yielded the least accurate results because they grow poorly; this problem deserves further exploration. The dominant species in polymicrobial was correctly identified in 81.2% of cases. In addition, to the best of our knowledge, we were the first to characterize the RMIs of rare bacteria that failed to grow on routing plates (including Methylobacterium, *Streptococcus salivarius thermophilus, Staphylococcus saccharolyticus*, and *Campylobacter fetus*). The RMI accuracy reached 100% for these species (*n* = 4), although the sample size was small, and should be increased for an accurate evaluation. The accurate and early detection of salmonella is important for the control and prevention of hospital infections.

A variety of studies have focused on the resistance determinants and activities of resistant enzymes (Majcherczyk et al., 2006; Grundt et al., 2012; Hrabák et al., 2012; Chan et al., 2015), but the obtained data were relatively scarce and unable to provide accurate MIC values to clinicians (Idelevich et al., 2014; Machen et al., 2014). Unlike the former methods, the Vitek AST system is highly sensitive and well-standardized. In this study, the category agreements of GN bacteria, *Staphylococcus* and *Enterococcus* spp., *Streptococcus* spp., and fungi reached 96.77, 93.5, 98.57, and 95.56%, respectively, similarly to previous studies (Romero-Gómez et al., 2012; Wimmer et al., 2012). The percentage of major and very major errors was low among the 3349 bacteria/antimicrobial combinations. In a RMAST of GN bacteria, most of the VMEs (0.49%, *n* = 9) occurred in amikacin, gentamicin, cefazolin, cefotetan, cefepime, and piperacillin-tazobactam, as observed for *E. coli* and *P. aeruginosa* in a previous study (Wimmer et al., 2012). Moreover, the present study obtained VMEs from *K. pneumoniae* and other non-fermenters. In the RMAST of *Staphylococcus* spp., most of the VMEs (*n* = 26) occurred in the presence of gentamicin, levofloxacin, clindamycin, and sulfamethoxazole/trimethoprim. It is worth mentioning that the VITEK ATB AST system could read the data automatically or semi-automatically. The automatic interpretation of low-growth streptococcal and fungal species could be supplemented by manual assistance. Thus, the RMAST category agreement of fungi is obviously higher in our study than in previous study (Idelevich et al., 2014). Importantly, the RMAST category agreement of *C. neoformans* reached 100%.

Accurate RMI/RMAST results are useful for optimizing a therapeutic regimen. First, the RMI and RMAST results are returned at least 18–36 h earlier than the final reports. Notably, the RMI of rare bacteria that failed to grow on routing plates might be advanced by 66.5 h. Second, the RMI results may accelerate the installation antibiotic therapy of BSIs caused by *S. aureus* (8.45%), *S. lugdunensis* (not detected in the present study) and *Listeria monocytogenes* (0.82%), which due to morphology consistent with the contaminated bacteria and gram stain are indistinguishable. The RMI also hastens the modification of empirical antibiotic treatment in cases showing intrinsic resistance (1.86% of cultures in the present study). From another viewpoint, 3.71% of the cultures indicated cessation of antibiotic therapy. Early recognitions of these contaminants would avoid wastage of medical resources. Third, the RMAST results provide a variety of accurate MIC values, by which clinicians can choose drug multi-directionally and estimate therapeutic doses precisely. In the present RMAST results, 13.44% of the cases showed resistance to last-resort antibiotics (such as vancomycin, linezolid, and carbapenem), further alerting clinicians to adjust the treatment or initiate essential combination therapy. In contrast, 51.56% of the bacteria were susceptible to third-generation cephalosporin and carbapenem (GN bacteria) or vancomycin and linezolid (GP bacteria), suggesting that antibiotic therapy might be de-escalated in patients infected by these organisms.

In this study, we theoretically examined the utility of RMI/RMAST in optimizing therapeutic regimens. We did not conduct a practical investigation. Several studies (Martiny et al., 2013; Perez et al., 2013) prospectively demonstrated that rapid pathogen identification and antimicrobial stewardship reduces the hospital length of stay and total costs. However, these studies were limited to BSI caused by GN bacteria. Therefore, we tentatively propose a protocol that establishes the control and intervention groups and detects bacterial, yeast and fungal infections in diverse sterile body fluids. The impact of RMI/RMAST in decreasing the hospital length of stay, cost, and antibiotic selective pressure deserves further investigation.

## Conclusions

The combined MALDI–TOF MS and Vitek AST system could obtain a rapid, accurate, reliable identification, and ASTs reports. The RMI and RMAST results not only reduce the turnaround time of the patient, but also guide clinicians whether a patient's antibiotic treatment should be accelerated, ceased or de-escalated, and adjusted the essential drugs modification for an optimized therapy.

## Ethics statement

This study was approved by the ethics committee of Huashan Hospital, Shanghai Medical College, Fudan University, Shanghai, People's Republic of China (protocol HS-H-2014-0213). All subjects provided written informed consent before their inclusion in the study.

## Authors contributions

YT and BZ performed all experiments. BW assisted in antimicrobial susceptibility testing. ML, YL, YT, and BZ conceived the study and analyzed the results. ML and YL supervised the study and wrote the manuscript. All authors read and approved the final manuscript.

## Funding

This study was supported by the National Natural Science Foundation of China (grants 81322025, 81171623 and 81371875), Outstanding Young Talent Plan of Shanghai (XYQ2011039), and Shanghai Shuguang Talent Project (12SG03).

### Conflict of interest statement

The authors declare that the research was conducted in the absence of any commercial or financial relationships that could be construed as a potential conflict of interest.

## Abbreviations

A list of abbreviations is shown in Table S2 in Supplementary Material.
