# Supplementary material for: Rapid Identification and Multiple Susceptibility Testing of Pathogens from Positive-Culture Sterile Body Fluids by a Combined MALDI-TOF Mass Spectrometry and Vitek Susceptibility System
Source: Front Microbiol. 2016 Apr 20;7:523. doi: 10.3389/fmicb.2016.00523 (PMC4837149; doi:10.3389/fmicb.2016.00523)
Supplement: Supplementary file 1 [file DataSheet1.docx]

**Table S1 The constitution and RMI** [**accuracy**](javascript:void(0);) **of the sterile body fluid positive cultures**

|  |  | **No/RMI** [**accuracy**](javascript:void(0);) **(%)** | | | | | | | | | |
| --- | --- | --- | --- | --- | --- | --- | --- | --- | --- | --- | --- |
| **Species** | **Total** | **Blood** | | **Csf** | | **Ascitic fluid** | | **Vitreous fluid** | | **Eye tissue** | |
| **Aerobic GN bacteria** | **189** |  |  |  |  |  |  |  |  |  |  |
| [*Enterobacteriaceae*](javascript:void(0);) | 134 | 130 | (100) |  |  | 4 | (100) |  |  |  |  |
| *Acinetobacter* spp | 28 | 28 | (100) |  |  |  |  |  |  |  |  |
| *Non-fermenters* | 27 | 26 | (92.3) | 1 | (100) |  |  |  |  |  |  |
| **Aerobic GP bacteria** | **203** |  |  |  |  |  |  |  |  |  |  |
| *Staphylococcus spp* | 139 | 127 | (89.0) | 5 | (100) | 4 | (100) | 3 | (100) |  |  |
| *Enterococcus spp* | 30 | 29 | (93.1) |  |  | 1 | (100) |  |  |  |  |
| *Streptococcus spp* | 21 | 15 | (66.7) |  |  | 6 | (83.3) |  |  |  |  |
| *Other GP bacteria* | 13 | 13 | (69.2) |  |  |  |  |  |  |  |  |
| **Fastidious bacteria** | **17** | **17** | **(11.8)** |  |  |  |  |  |  |  |  |
| **Fungi** | **37** |  |  |  |  |  |  |  |  |  |  |
| *Yeast* | 21 | 16 | (62.5) | 1 | (100) | 1 | (100) |  |  | 3 | (100) |
| *Cryptococcus spp* | 16 | 7 | (57.1) | 9 | (100) |  |  |  |  |  |  |
| **Anaerobic species** | **19** | **19** | **(94.7)** |  |  |  |  |  |  |  |  |
| **Bacteria failed to**  **grow on the routing**  **plates** | **4** | **4** | **(100)** |  |  |  |  |  |  |  |  |
| **Polymicrobial** | **16** | **16** | **(81.2)** |  |  |  |  |  |  |  |  |

a: RMI [accuracy](javascript:void(0);) was scored by the modified criterion (genus level).

**Table S2 The list of abbreviations**

|  | **abbreviations** |  | **abbreviations** |
| --- | --- | --- | --- |
| antimicrobial susceptibility tests | ASTs | brain heart infusion broth | BHI |
| rapid microbial identification | RMI | cerebrospinal fluid | CSF |
| rapid multiple AST | RMAST | Blood culture | BC |
| Gram negative | GN | McFarland | McF |
| Gram positive | GP | minimum inhibitory concentrations | MIC |
| bloodstream infection | BSI | very major error | VME |
| identification | ID | major error | ME |
| Matrix-assisted laser desorption/ionization time of flight mass spectrometry | MALDI–TOF MS | minor error | mE |


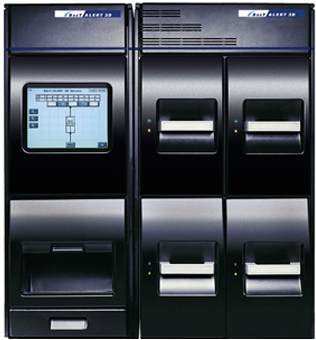


(a)


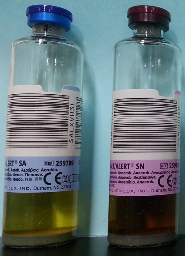


(b)


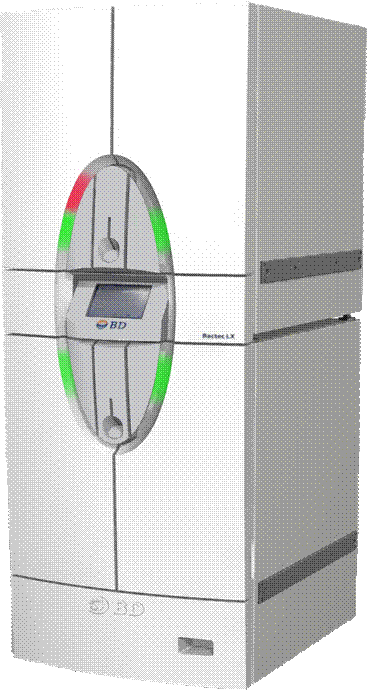


(c)


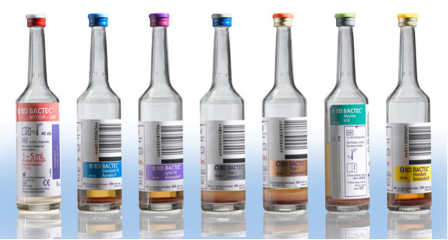


(d)

Figure S1: BC system and BC bottles. (a): BacT/Alert 3D, bioMérieux BC system; (b): BacT/Alert 3D, bioMérieux BC bottles; (c): BACTEC FX, Becton Dickinson BC system; (d): BACTEC FX, Becton Dickinson BC bottles.
